# Effect of Ebola virus proteins GP, NP and VP35 on VP40 VLP morphology

**DOI:** 10.1186/1743-422X-3-31

**Published:** 2006-05-23

**Authors:** Reed F Johnson, Peter Bell, Ronald N Harty

**Affiliations:** 1 Department of Pathobiology, School of Veterinary Medicine, University of Pennsylvania, 3800 Spruce St., Philadelphia, PA 19104, USA; 2 Gene Therapy Program, Department of Pathology and Laboratory Medicine, School of Medicine, University of Pennsylvania, Philadelphia, PA 19104, USA

## Abstract

Recently we described a role for Ebola virus proteins, NP, GP, and VP35 in enhancement of VP40 VLP budding. To explore the possibility that VLP structure was altered by co-expression of EBOV proteins leading to the observed enhancement of VP40 VLP budding, we performed density gradient analysis as well as electron microscopy studies. Our data suggest that VP40 is the major determinant of VLP morphology, as co-expression of NP, GP and VP35 did not significantly change VLP density, length, and diameter. Ultra-structural changes were noted in the core of the VLPs when NP was co-expressed with VP40. Overall, these findings indicate that major changes in morphology of VP40 VLPs were likely not responsible for enhanced budding of VP40 VLPs in the presence of GP, NP and/or VP35.

## Introduction

Ebola and Marburg viruses are members of the *Filoviridae *family of the order *Mononegavirales*. Both viruses are associated with recurrent outbreaks of deadly hemorrhagic fevers with mortality rates as high as 90% [1,2]. Currently, there are no approved vaccines, nor treatments for Ebola virus (EBOV) infection. A better understanding of the molecular aspects of EBOV replication will be necessary for successful development of specific treatments for EBOV infection.

Ebola virus matrix protein, VP40, is the major virion protein and plays an essential role in virus assembly and budding [3,4]. VP40 buds from the cell surface forming virus-like particles (VLPs). VLP budding is mediated by viral L-domains present in the N-terminus of the protein, which interact with host factors such as Nedd4 and TSG101, leading to VLP release [3-7]. It is hoped that investigations into the mechanisms of VP40 VLP budding will lead to possible vaccines and therapeutics that will block late stages of the virus life-cycle.

Recent evidence suggests that co-expression of other EBOV proteins will enhance VP40 VLP budding [8,9]. For example, co-expression of VP40+GP+NP enhanced VP40 release approximately 40-fold over that observed for VP40 alone [9]. We have also demonstrated that VP35 interacts with VP40, is enclosed within VP40 VLPs, and functions to specifically package the EBOV 3E-5E minigenome into VLPs [10]. Currently, the mechanism by which EBOV proteins enhance VP40 budding is unclear, as is their affect on VLP morphology. Thus, we are interested in examining VLPs that contain combinations of VP40, GP, NP, and VP35 to determine whether co-expression of different EBOV proteins affects density, length, diameter, and overall morphology. Investigating the morphology of EBOV VLPs may give us insight into the mechanism by which EBOV proteins contribute to the observed enhancement of VLP budding.

Early EBOV reports suggest the virus particle is 970 nm in length and 80 nm in diameter with a density of 1.14 g/mL [11-13]. Since EBOV is a bio-safety level 4 pathogen, alternate means to study its properties have been developed. The most commonly used method to study EBOV proteins is transfection and co-expression of plasmids coding for individual viral proteins. Using this approach, Bavari et al. have demonstrated that co-expression of VP40 and GP yielded VLP particles 50–70 nm in diameter and 1–2 μm in length [13], while Jasenosky et al. determined the VP40 VLP particle density to be 1.11–1.13 g/ml [4]. In addition, Noda et al. demonstrated that GP formed 10 nm long spikes on the surface of VP40 VLPs, and VLPs were found to be 10 μm in length.

In this report, we performed sucrose density gradient sedimentation, electron microscopy (EM), and protease protection assays on VLPs from cells transfected with combinations of VP40, GP, NP, and/or VP35. We demonstrate that there are minimal changes in VLP density, diameter, and wall thickness with co-expression of other viral proteins. Statistically significant differences were found in measurements of wall thickness between VP40 VLPs and VP40+VP35 VLPs. Lastly, NP was packaged within VP40+NP VLPs, and VLP morphology was altered when NP was co-expressed with VP40.

## Results

### NP is packaged within VP40 VLPS

**Figure 1:**
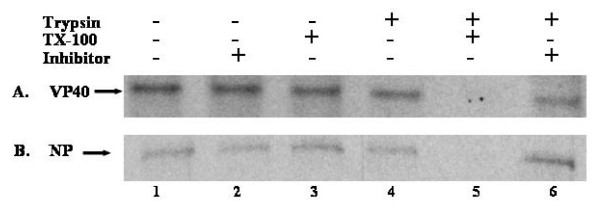
Protease Protection Assay of VLPs containing NP and VP40. VLP pellets were split into six fractions and treated with STE buffer (lane 1), soybean trypsin inhibitor (lane 2), Triton X-100 (lane 3), Trypsin (lane 4), Trypsin and Triton X-100 (lane 5), Trypsin and soybean trypsin inhibitor (lane 6). The fractions were divided equally, and viral proteins were immunoprecipitated with α-VP40 (Panel A), or α-NP (Panel B). Samples were resolved by SDS-PAGE and detected by autoradiography.

We have demonstrated previously that NP enhances VP40 VLP budding 3.5 fold over VP40 alone, but did not demonstrate that NP was packaged within VP40 VLPs [9]. To prove that NP is packaged within VP40 VLPs, protease protection assays were performed. Similar experiments have been performed with VP35 to demonstrate that VP35 is also packaged within VP40 VLPs [10]. Human 293T cells were transfected with pCAGGS vector alone, VP40, NP, or VP40+NP. Purified VLPs were divided into six equal fractions. As reported previously, VP40 was only digested in the presence of both Triton X-100 and trypsin (Fig 1A, Lane 5) [6]. Similarly, we found that NP was degraded completely only in the presence of both Triton X-100 and trypsin (Fig. 1B, lane 5). Treatment with trypsin alone was insufficient to digest NP (Fig 1B, lane 4), indicating that NP is packaged within VP40 VLPs. It should be noted that NP was unable to bud from cells as a VLP when expressed alone in mammalian cells [9].

### VP40 is the major determinant of EBOV VLP density

VLP density was determined by centrifugation of VLPs purified from transfected 293T cells over 20% sucrose in STE, followed by centrifugation on a 20–50% sucrose gradient for 14 hours. Five hundred microliter fractions were collected and analyzed for density using a refractometer. Fractions positive for VP40 as determined by Western blot were converted to density. Table 1 lists the densities for each combination of EBOV proteins expressed, and these data represent an average of 7 independent experiments.

**Table 1:** VLP Density on 20–50% sucrose in STE.

	**Density g/mL**	**Standard Deviation**	**pNUM **(t-test compared to VP40)
VP40 (n = 7)	1.102	0.0262	
VP40+VP35 (n = 7)	1.125	0.003	0.5
VP40+VP35+NP (n = 7)	1.118	0.006	0.15
VP40+NP (n = 7)	1.102	0.035	0.34
VP40+GP (n = 7)	1.091	0.038	0.45
VP40+NP+GP (n = 7)	1.104	0.031	0.27

The average density of VP40 VLPs was 1.102 g/mL. Co-expression of VP35 and VP40 increased VLP density to that of 1.125 g/mL. Co-expression of VP40+VP35+NP yielded VLPs with a density of 1.118 g/mL, whereas VP40+NP VLPs had a density of 1.102 g/mL (Table 1). These data indicate a slight decrease in VLP density with the inclusion of GP to VP40 VLPs yielding a density of 1.091 g/mL. When NP was included with VP40+GP, the density increased to 1.104 g/mL; closer to that obtained with VP40 alone. It should be noted that no statistically significant differences were measured, and each VLP density measurement was well within the standard deviation of VP40 VLPs of 0.026 (Table 1). The density of the VLPs suggests that VP40 is the major determinant of density of the VLP particle. Jasenosky et al reported VP40 VLP density of 1.11 to 1.13 g/mL, which is close to EBOV observations (1.14 g/mL) [4,11,12]. Our findings suggest that VP40 determines density of the VLP particle, as co-expression of EBOV proteins did not significantly change VLP density.

### Electron microscopic analysis of EBOV VLPs

**Figure 2:**
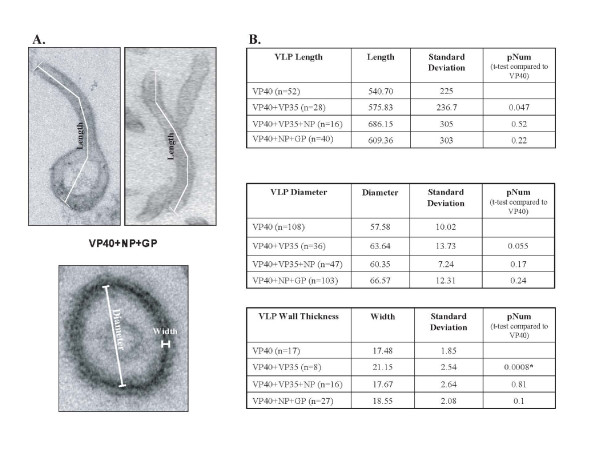
A. Electron micrographs of individual VLP particles demonstrating the dimensions measured. B. Summary measurements of VLP Length, Diameter, and Wall Thickness. The data demonstrate that measured differences between VLPs are not statistically significant, except for VP40+VP35 VLPs. The (*) indicates statistically significant differences.

**Figure 3:**
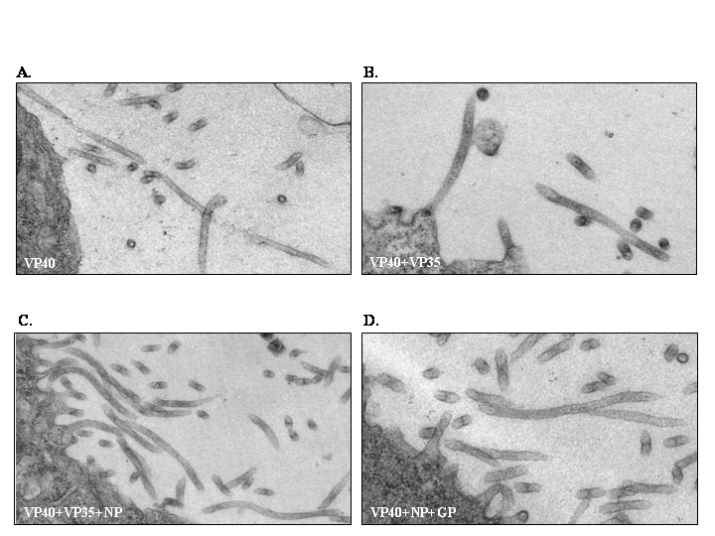
Electron micrographs for A) VP40 VLPs, B) VP40+VP35 VLPs, C) VP40+VP35+NP VLPs, and D) VP40+NP+GP VLPs.

Combinations of EBOV proteins (VP40, VP40+VP35, VP40+VP35+NP, VP40+NP+GP) were expressed in 293T cells, and samples were prepared for analysis by electron microscopy. Measurements of length, diameter, and wall thickness were taken for each set of VLPs, and these values were compared to those obtained for VP40 alone. Overall length of VLPs was measured (Fig. 2 and Fig. 3) resulting in an average length of 540.70 nm for VP40 VLPs, 575.83 nm for VP40+VP35, 686.15 nm for VP40+VP35+NP, and 609.36 nm for VP40+NP+GP. It should be noted that length measurements likely reflect a mixture of full and partial-length VLPs. We identified several particles that correlated with full-length measurements reported previously by Bavari et al. (1.0–2.0 μm) [13]. The largest overall length of 1277 nm was observed for VP40+NP+GP VLPs, while the smallest overall length of 204 nm was observed for VP40 alone.

VP40 VLPs had an average diameter of 57.58 nm, with slight increases for VP40+VP35 at 63.64 nm, VP40+VP35+NP at 60.35 nm, and VP40+NP+GP at 66.57 nm (Fig. 2). These data are consistent with diameters reported by Bavari et al of 50–70 nm and narrower than that reported for EBOV of 80 nm [13]. These data support a role for VP40 as the major component that determines VLP length and diameter. Co-expression of NP, GP, and/or VP35 did not significantly alter VLP diameter. (Fig. 2.)

**Figure 4:**
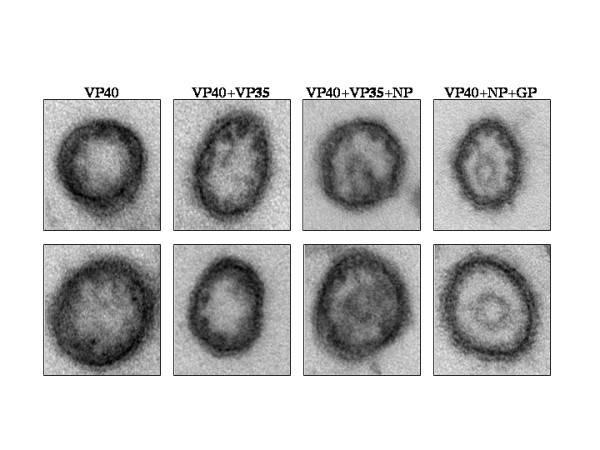
Representative examples of VLPs in cross section. Note the appearance of a bull's-eyes structure within VLPs containing NP.

A morphological difference was observed following examination of cross-sections of VLPs expressing VP40 alone vs. those expressing additional EBOV proteins. For example, VP40 and VP40+VP35 VLPs had disorganized centers, while VLPs which included NP possessed a "bull's-eye" appearance (Fig. 4). In addition, the region around the membrane appeared more uniform in VLPs that included NP (Fig. 4). Next, we measured the wall thickness of VLPs in cross section (see Figure 2 for detailed description of what we defined as diameter and "wall thickness"). The average measurement of wall thickness was 17.48 nm for VP40 VLPs, 21.15 nm for VP40+VP35, 17.67 nm for VP40+VP35+NP, and 18.55 nm for VP40+NP+GP (Figs. 2 and 4). A statistically significant difference was found between VP40 and VP40+VP35 with a pNum value of 0.008.

## Conclusion

Overall, expression of GP, NP and/or VP35 with VP40 did not drastically affect VLP density or diameter. However, wall thickness increased slightly with co-expression of VP35 and VP40. We also observed packaging of NP within VP40+NP VLPs and altered VLP morphology when NP was co-expressed with VP40+GP or VP40+VP35. These data suggest that VP40 is the major determinant of VLP morphology. These data support prior findings by Noda, Bavari, and Jasenosky [4,5,13], and expand upon these findings by incorporation of NP and VP35 into VLPs.

Our data demonstrate that VLP density increased with the inclusion of the nucleocapsid proteins NP and VP35 (NP+VP35 – 1.118 g/mL, VP35 – 1.125 g/mL) suggesting that these VLPs may be more organized and more closely resemble authentic virus particles. In contrast, when GP was co-expressed with VP40, a slight decrease in density was observed (1.102 vs. 1.091). This may be due to a change in morphology of the particle and a resulting retardation in the sucrose gradient by extension of GP from the VLP surface [5]. VP40+NP+GP and VP40+NP VLPs had densities roughly equal to that of VP40 alone, with VP40+NP particles being slightly denser. These findings suggest that the contribution of NP to density of the particle offsets the decrease in density by GP. Statistical analysis suggests that the densities are within one standard deviation of the density of VP40 alone. It is also important to point out that densities observed here and by Jasenosky et al. are slightly less than that observed for mature virions (1.14 g/mL). This difference could be due to complete incorporation of nucleocapsids and less variability in particles produced due to infection vs. transfection.

Morphometric analyses suggest that there are differences in the lengths of the VLP particles (Fig. 2); however, length measurements likely vary due to the possibility that complete VLP particles were not consistently measured. Indeed it is likely that only a portion of a VLP was measured due to the VLP exiting the plane of section being inspected. Analysis by EM also suggests that there were slight differences in VLP diameter with co-expression of the nucleocapsid components VP35 and NP (Fig. 2). These differences were not found to be statistically significant. Overall particle diameter was as follows: 57.58 nm for VP40 VLPs, 63.64 nm for VP40+VP35 VLPs, 60.35 nm for VP40+VP35+NP VLPs, and 66.57 nm for VP40+NP+GP VLPs. The increase in diameter of particles containing VP35 correlates with cross-sectional observations of wider particles with no organized centers (Fig. 4). Additionally, morphological differences were evident in VLPs expressing NP (bull's-eye, Fig. 4). The formation of the bull's-eye within the VP40+VP35+NP VLPs and VP40+NP+GP VLPs may indicate that NP self-aggregated (as is seen with Marburg NP [14]) resulting in electron-dense material within VLPs. VP40+VP35+NP VLPs were also slightly more narrow than those containing VP40+VP35, suggesting an organization of the core of the virus imparted by NP+VP35 nucleocapsid formations [15]. The "bull's-eye" present in VP40+GP+NP VLPs along with the results of the protease protection assay suggest that NP is packaged within VLPs.

The wall thickness of the VLPs containing EBOV proteins was found to vary. Indeed, statistical analysis indicated a significant difference between VLPs containing VP40 alone vs. those containing VP40+VP35. Gross inspection of the particles indicated a less uniform appearance and thickening of the VLP wall in VP40+VP35 VLPs. When NP was co-expressed, the wall appeared more uniform around the circumference of the VLP, suggesting that NP has an organizing effect during the formation of VLPs.

In sum, our data suggest that VP40 is the major determinant of VLP density, and co-expression of NP, GP, and/or VP35 had minor affects on VLP morphology. It will be of interest to use immuno-EM to more precisely map the location of EBOV proteins within VLPs. Thus, the enhancement of VP40 release following co-expression of additional EBOV proteins [8,9] is unlikely to be due to gross changes in VLP morphology. One possibility is that enhancement of VP40 release may be due to modifications of the cellular architecture, or effects on host protein function as a result of EBOV protein expression [16].

## Materials and methods

### Cells, plasmids, and antibodies

293T cells were maintained in Dulbecco Modified Eagles Media (DMEM) 10% FBS, 5% CO_2_. pCAGGS, pCAGGS VP40, pCAGGS NP, and pCAGGS GP have been described previously [9]. The pCAGGS VP35 construct was provided by Chris Basler (Mount Sinai, New York) and contains an influenza hemagglutanin (HA) epitope tag at the amino terminus of VP35. Antibodies to VP40 were provided by Roland Grunow (Marburg, Germany). Antibodies to NP were provided by Jason Paragas (Fort Detrick, USAMRIID). Antibodies to GP were provided by Paul Bates (Univ. of Penn). HA antibody was obtained from Roche.

### Protease protection assay

VLP budding assays were performed as described previously [4,6,17] with minor modifications as follows. 293-T cells on 100 mm tissue culture dishes (Corning) were transfected with 10 μg of pCAGGS, pCAGGS VP40, or pCAGGS NP+ pCAGGS VP40 plasmids using Lipofectamine (Invitrogen). 1600 μCi of [^35^S]Met/Cys (Perkin Elmer) was added to each 100 mm dish 24 hours post-transfection. At thirty hours post transfection, culture medium was clarified at 1500 rpm for 10 minutes, layered over a 20% sucrose in STE buffer (0.01 M Tris-HCl [pH 7.5], 0.01 M NaCl, 0.001 M EDTA [pH 8.0]) cushion, and VLPs were purified at 36,000 rpm for 2 hours at 4°C. VLPs were suspended in 400 μL of STE buffer and divided into six, 60 μL fractions. The fractions were treated with either 6 μL of STE buffer, 3 mg/mL soybean trypsin inhibitor (Roche Biochemicals), 1% Triton X-100 (Fisher), 0.1 mg/mL trypsin (Promega), 1% Triton X-100 and 0.1 mg/ml trypsin, or 0.1 mg/ml trypsin and 3 mg/mL trypsin inhibitor. All fractions were incubated at room temperature for 30 minutes followed by the addition of 25 μL of soybean trypsin inhibitor at a concentration of 100 mg/mL to quench the reactions. Each fraction was lysed in RIPA buffer (50 mM Tris [pH 8.0], 150 mM NaCl, 1.0% NP-40, 0.5% deoxycholate, 0.1% sodium dodecyl sulfate [SDS]) in a final volume of 500 μL. Proteins were immunoprecipitated with appropriate antibodies, and resolved by SDS-Polyacrylamide gel electrophoresis (PAGE). As a control for protein expression, the transfected cells were lysed in RIPA buffer and split into equal fractions. Immunoprecipitated proteins were resolved by SDS-PAGE, and visualized by autoradiography.

### VLP density analysis

VLP budding assays were performed as described previously [4,6,17] with minor modifications as follows. 293T cells on 100 mm tissue culture dishes (Corning) were transfected with 10 μg of pCAGGS, pCAGGS VP40, pCAGGS VP35+pCAGGS VP40, pCAGGS NP+pCAGGS VP40, or pCAGGS VP40+pCAGGS GP, or pCAGGS VP40+pCAGGS NP+pCAGGS VP35, or pCAGGS VP40+pCAGGS NP+pCAGGS GP plasmids using Lipofectamine (Invitrogen). VLPs were purified over 20% sucrose as previously described [4,5,9]. VLPs were resuspended in 500 μL STE buffer and overlayed onto a 20–50% sucrose gradient prepared in STE buffer. VLPs were centrifuged at 36,000 × G for 14 hours and 0.5 mL fractions were collected for each sample. An equal portion of each fraction was run on a 10% SDS-PAGE gel, transferred and probed with anti-VP40 antibody. Transfected cells were lysed in RIPA buffer and electrophoresed over a 10% SDS-PAGE gel, transferred and probed with anti-VP40, anti-GP, anti-NP, and anti-HA to ensure that all viral proteins were expressed.

### Electron microscopy and morphometry

Human 293T cells on 6-well tissue culture dishes (Corning) were transfected with 1 μg of pCAGGS, pCAGGS VP40, pCAGGS VP40+pCAGGS VP35, pCAGGS VP40+pCAGGS NP, or pCAGGS VP40+pCAGGS GP, or pCAGGS VP40+pCAGGs NP+pCAGGS VP35, or pCAGGS VP40+pCAGGS NP+pCAGGS GP plasmids using Lipofectamine (Invitrogen). Transfected cells were harvested and centrifuged at 3000 rpm in a microcentrifuge for 5 min to form a loose pellet. Cell pellets were then fixed overnight in 2.5% glutaraldehyde, 2% paraformaldehyde in 0.1 M sodium cacodylate buffer (pH 7.3), washed in cacodylate buffer, and incubated in 2% OsO_4 _for 2 hours. After washing with water, samples were stained overnight in 0.5% uranyl acetate, washed again, dehydrated through a graded ethanol series and propylene oxide, and finally embedded in resin (LX-112, Ladd Research Industries). Ultrathin sections (80 nm) were stained with uranyl acetate and lead citrate according to standard protocols and examined with a Philips CM-100 transmission electron microscope equipped with a KeenView digital camera system. Morphometric measurements were performed on digital images using AnalySIS software (Soft Imaging System, Lakewood, CO).
